# Application of Stable Isotopes and Multi Elemental Fingerprints to Verify the Origin of Premium Chinese Hainan Bananas

**DOI:** 10.3390/foods14040554

**Published:** 2025-02-07

**Authors:** Yurong Huang, Hanyi Mei, Yongzhi Zhang, Mingyue Wang, Zhibo Huan, Jing Nie, Karyne M. Rogers, Bayan Nuralykyzy, Chunlin Li, Yuwei Yuan

**Affiliations:** 1 College of Agriculture and Animal Husbandry, Qinghai University, Xining 810016, China; 19517994504@163.com; 2 State Key Laboratory for Managing Biotic and Chemical Threats to the Quality and Safety of Agro-Products, Zhejiang Academy of Agricultural Sciences, Hangzhou 310021, China; meihy@zaas.ac.cn (H.M.); zhangyz@zaas.ac.cn (Y.Z.); niej@zaas.ac.cn (J.N.); karynerogers@gmail.com (K.M.R.); baiana2020@nwafu.edu.cn (B.N.); 3 Key Laboratory of Information Traceability for Agricultural Products, Ministry of Agriculture and Rural Affairs of China, Institute of Agro-Products Safety and Nutrition, Zhejiang Academy of Agricultural Sciences, Hangzhou 310021, China; 4 Analysis and Testing Center, Chinese Academy of Tropical Agricultural Sciences, Haikou 571101, China; hkwmy0815@163.com (M.W.); huanzhibo@163.com (Z.H.); 5 School of Geography, Environment and Earth Sciences, Victoria University of Wellington, Wellington 6012, New Zealand

**Keywords:** *Musa nana* Lour., *δ*^13^C, *δ*^15^N, *δ*^18^O, *δ*^2^H, elements, chemometrics, PLS-DA, model, traceability

## Abstract

China is the world’s largest consumer and second largest producer of bananas. This strong domestic demand consistently provides a reliable income for Chinese banana growers. The geographical origin of food is usually associated with product quality and safety, and this is especially noted for Hainan origin-labeled bananas, which are grown offshore on China’s largest tropical island. Hainan banana is recognized as a premium variety within China’s banana market, but there have been recent impacts on branding, profits, and a reduction in income for banana farmers due to the fraudulent in-market substitution of non-Hainan bananas. In this study, stable isotope and elemental chemometric models were used to differentiate bananas grown in Hainan province (HN) from non-Hainan provinces (NHN). The results showed that HN bananas had a specific isotopic and elemental fingerprint compared to NHN bananas. Bananas sampled from HN and NHN regions showed significant differences in *δ*^13^C values (HN: −22.2‰ to −27.7‰, NHN: −22.3‰ to −24.3‰), Al content (HN: 0.00 mg/kg to 0.10 mg/kg, NHN: 0.00 mg/kg to 0.02 mg/kg), Na content (HN: 0.00 mg/kg to 0.09 mg/kg, NHN: 0.00 mg/kg to 0.07 mg/kg), and other elements (*p* < 0.05). Overall, 14 key variables reflecting climate and soil properties were selected from a group of 53 variables to improve a partial least squares discriminant analysis (PLS-DA) chemometric model. The discrimination accuracy of the test set increased from 84.60% to 90.93% after variable reduction. The use of stable isotopes and elements combined with PLS-DA models provided an effective method for distinguishing Chinese HN bananas from NHN bananas and would be useful as a screening or regulatory tool to confirm instances of origin fraud.

## 1. Introduction

Banana (*Musa nana* Lour.) is widely cultivated in tropical and subtropical regions such as Asia. Low in fat and sugar but high in minerals, fiber, and vitamins, bananas make an ideal snack, and are very popular with consumers [1]. According to the United Nations Food and Agriculture Organization (FAO) data in 2019, banana plantations account for about 5,158,600 hectares globally. In China, banana plantations cover 358,900 hectares, with an annual production of about 12 million tons, making China the world’s second-largest banana producer [2]. China’s main banana production regions are Hainan, Guangdong, Guangxi, Yunnan, and Fujian provinces [3]. Excluding Hainan province, these four main banana-producing regions account for about 84% of the main banana-producing areas in China and about 83% of the production. Specific information such as the banana output value of the four provinces is shown in Table S1, and this study focuses on the above five main banana-producing areas.

Among all the bananas grown in China, Hainan banana (originating from Hainan Province) is recognized by consumers as a high-quality banana product (Figure S1). Hainan banana (*Uvaria calamistrata* Hance) belongs to the Annonaceae family, and it is popular due to its aromatic taste, large size, bright color, and richness in vitamins and minerals. It also has a very low sugar content, which is beneficial for people who need to control their blood sugar levels. At the same time, it is harvested abundantly throughout the year and provides strong economic returns to growers. According to government statistics in 2021, the total output of bananas in Hainan Province of China reached 1.16 million tons, with an output value of 3.5 billion yuan [4].

Hainan Province is located in the southernmost part of China, on an offshore tropical island. The island’s area accounts for 42.5% of China’s tropical agricultural land, and its location results in high sunshine hours, temperature, and water availability—all favorable growing conditions for a flourishing banana industry [5]. In recent years, there have been some inconsistent phenomena in the market, such as non-Hainan-produced bananas sold under the Hainan banana brand [6] and food safety incidents [7], which have affected the reputation of the banana brand in Hainan Province, reducing the economic growth of the industry and the income of banana growers.

Origin verification technology offers a potential tool that could secure the Hainan banana brand [8] and protect the economic interests of Hainan’s banana industry and banana farmers. In addition, this technology could ensure consumers do not pay higher prices for geographically mislabeled bananas [9]. As a result, there is a strong interest in developing banana origin traceability and identification techniques [10].

Stable isotopes are naturally occurring markers capable of carrying information about environmental factors in biological and geological materials. In nature, organisms continuously exchange isotopes with the external environment and are related to geographical factors (altitude, latitude, and longitude), climatic conditions (temperature, precipitation, and light intensity), and physiological processes (photosynthesis, respiration, and transpiration) [11,12]. The elemental contents of plants and organisms are related to different types of soil, available nutrients, and water, which change spatially, resulting in different elemental fingerprints according to geographical origin [13,14]. Currently, isotope and multi-element investigations have already achieved favorable fruit origin traceability results for apples [15], durian [16], pears [17], and other fruits. For example, Wen et al. [18] used inductively coupled plasma mass spectrometry (ICP-MS) to determine 167 elements in 38 jujube samples and classify the major jujube-producing areas in China. Identifying the origin of jujube by chemometric multi-element fingerprint analysis is a practical and accurate approach and provides guidelines for establishing a traceability system for the origin of other fruits. Zhang [19] used element analysis and stable isotope mass spectrometry (EA-IRMS) to measure multiple stable isotopes in cherry pulp and pits. Partial least squares discriminant analysis (PLS-DA) was applied to the data, which preliminarily verified the use of stable isotopes to correctly determine the origin of sweet cherries. Li et al. [20] explored the feasibility and effect of elemental fingerprints to trace the origin of blueberries. This study achieved an accuracy rating of 95.8% and concluded that elemental fingerprints were useful origin indicators. Bin et al. [21] utilized stable isotopes and element stoichiometry techniques to study the geographical origin of melons in the Xinjiang region of China, effectively ensuring the integrity of Hami melon’s provenance and enhancing market confidence.

The aim of this study was to use stable isotopes and elemental fingerprints of bananas sampled from Hainan (HN) and non-Hainan (NHN) regions in China (Yunnan, Guangxi, Guangdong, Fujian) and to establish a model to classify the geographical origin of bananas using chemometric methods. This research aims to identify the unique isotopic and elemental characteristics of bananas from different origins in China, potentially offering a validation tool to protect the economic interests of the Hainan banana industry.

## 2. Materials and Methods

### 2.1. Materials

**Figure 1:**
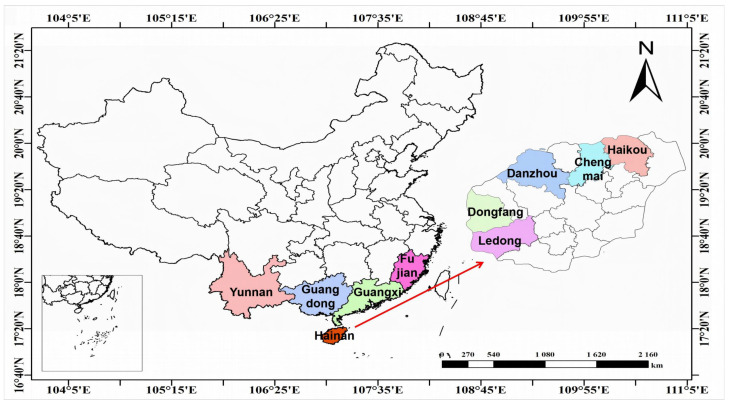
Location of banana sampling regions in China.

A total of 123 banana samples were collected from the main planting regions in the south of China, representing 16 cities from five provinces: Hainan (*n* = 75), Yunnan (*n* = 19), Guangxi (*n* = 9), Guangdong (*n* = 9), and Fujian (*n* = 11) (Figure 1). Five cities (counties) from Hainan Province were sampled: Chengmai (*n* = 23), Danzhou (*n* = 25), Haikou (*n* = 7), Dongfang (*n* = 14), and Ledong (*n* = 6) to gain a representative banana fingerprint from Hainan Province.

Banana samples were collected directly from orchards to ensure a known origin. In each city or county, 4–7 bunches of bananas were collected from each orchard, and 2–3 bananas were selected from each bunch to form a composite sample. Specific collection information is shown in Table S2, and the sampling locations are shown in Figure 1.

### 2.2. Sample Pre-Treatment

Two or three bananas were peeled and homogenized together in a blender (HR2168, Philips, Shanghai, China) with 500 mL of distilled water. Subsequently, 50 g of the homogenized sample was transferred into a 50 mL centrifuge tube, sealed with film, and frozen for 2 h. After puncturing the film, the sample was freeze-dried (SCIENTZ-18, Ningbo, China) to a constant weight, with a drying duration of approximately 30 h. The dried sample was then powdered using a grinder (HR2864, Philips, Shanghai, China), sieved through a 100-mesh screen, and stored in 15 mL sealed tubes in a desiccator away from light.

### 2.3. Stable Isotope Analysis

(1) Stable carbon and nitrogen isotopes

Around 5 mg of powdered banana was weighed and wrapped in a tin foil cup (9 × 5 mm) in duplicate and placed in an elemental analyzer (Elementar Vario PYRO Cube, Elementar, Langenselbold, Germany) along with blanks and reference materials. The carbon and nitrogen elements in the samples were converted into pure CO_2_ and N_2_ gases and then admitted into an isotope ratio mass spectrometer (IRMS, Isoprime 100, Isoprime, Manchester, UK). Analytical conditions: the helium purge flow rate of the elemental analyzer was 230 mL/min; the oxidation and reduction furnace temperatures were 1150 °C and 850 °C, respectively, and the helium flow rate of the carrier gas into the mass spectrometer was 100 mL/min.

(2) Stable hydrogen and oxygen isotopes

A high-temperature pyrolysis elemental analyzer-isotope ratio mass spectrometer (HT/EA-IRMS, Elementar, Germany) was used to determine the hydrogen and oxygen isotopes. A 0.5 mg sample of powdered banana was weighed and wrapped in a silver cup (8 × 5 mm) in triplicate and placed in an elemental analyzer along with blanks and reference materials. The samples were pyrolyzed at high temperature in a combustion furnace, and the residual gases were admitted into the isotope mass spectrometer for analysis. Analytical conditions: the encapsulated samples and reference materials were freeze-dried at −60 °C for 48 h to remove adsorbed (exchangeable) water, then equilibrated in the laboratory for 5 days while exposed to local atmospheric conditions before analysis. The pyrolysis temperature was 1450 °C and the reference gases were H_2_ and CO.

(3) Isotope calculation and reference materials

Stable isotope values are calculated using the following equation:

*δ* ‰ = [(R_Sample_/R_Standard_) − 1]

where R_Sample_ is the abundance ratio of heavy to light isotopes in the measured sample, i.e., ^13^C/^12^C, ^15^N/^14^N, ^18^O/^16^O, ^2^H/^1^H, and R_Standard_ is the abundance ratio of heavy to light isotopes in the standards. The *δ* values are reported relative to Vienna Pee Dee Belemnite (V-PDB, International Atomic Energy Agency) for *δ*^13^C, Air for *δ*^15^N, and Vienna Standard Mean Ocean Water (V-SMOW, International Atomic Energy Agency, Vienna, Austria) for *δ*^18^O and *δ*^2^H.

Multi-point calibration was performed using commercially available stable isotope reference standard materials. These included: IAEA-CH-6 (sucrose, *δ*^13^C_V-PDB_ = −10.449 ± 0.033‰), IAEA-600 (caffeine, *δ*^13^C_V-PDB_ = −27.771 ± 0.043‰, *δ*^15^N_air_ = 1.0 ± 0.2‰), IAEA-601 (benzoic acid, *δ*^18^O_V-SMOW_ = 23.14 ± 0.19‰), IAEA-602 (benzoic acid, *δ*^18^O_V-SMOW_ = 73.35 ± 0.39‰), IAEA-N-2 (ammonium sulfate, *δ*^15^N_air_ = 20.3 ± 0.2‰), and IAEA-CH-7 (polyethylene, *δ*^2^H_V-SMOW_ = −100.3 ± 2.0‰), procured from the International Atomic Energy Agency (IAEA, Vienna, Austria); B2203 (*δ*^2^H_V-SMOW_ = −25.3 ± 1.1‰), B2155 (*δ*^15^N_air_ = 5.94 ± 0.08‰), B2174 (*δ*^13^C_V-PDB_ = −37.421 ± 0.017‰), were purchased from Elemental Micro-analysis, UK, and USGS55 (*δ*^2^H_V-SMOW_ = −28.20‰, *δ*^18^O_V-SMOW_ = 19.12‰) from the United States Geological Survey, Reston, VA, USA.

### 2.4. Elemental Analysis

Around 0.1 g of each dried banana sample was accurately weighed and added to a Teflon microwave digestion vessel. A 7.0 mL nitric acid solution was added, and the sample was digested for 12 h. Then, 1.0 mL of hydrogen peroxide was added to a microwave digestion apparatus (CEM Mars 5, CEM Inc., Matthews, NC, USA) to oxidize the organic matter. The subsequent digestion procedure consisted of the following steps: The temperature was elevated to 120 °C and held for 2 min; then increased to 150 °C and held for an additional 2 min; then raised to 180 °C and held for 2 min; finally, heated to 200 °C and held for a further 20 min. Next, the sample was heated on an acid evaporation instrument (CEM Inc.) for 30 min to dry off the nitric acid from the sample. Finally, the digested solution was transferred to a plastic centrifuge tube, diluted to a volume of 10.0 mL with ultrapure water, mixed well, and filtered through a 0.45 μm filter before analysis using an ICP-MS (Thermo Fisher, Waltham, MA, USA). Multi-element determination was monitored, and instrumental drift was corrected using an internal standard solution (1 ng/mL) containing 49 elements analyzed in conjunction with samples in this study. This assay method is referenced in a previous publication [22] and the certified internal standard reference material (Astragalus-GBW10028) used to control experimental accuracy was obtained from the National Research Center for Certified Reference Materials (Beijing, China).

The operational parameters of the ICP-MS are as follows: The plasma temperature was maintained at 6000 K, with a peristaltic pump flow rate of 1 mL/min. The radio frequency (RF) power was set at 1550 W, and the sampling depth was 8 mm. The plasma gas flow rate was 15.0 L/min, the carrier gas flow rate was 100 L/min, and the diluent gas flow rate was 0.05 L/min. The sample introduction cone was composed of nickel, and the flow rates for the pool reaction gases such as helium (He), hydrogen (H_2_), oxygen (O_2_), and ammonia (NH_3_) were 4.5, 4.0, 1.5, and 1.0 mL/min, respectively.

### 2.5. Statistical Analysis

Mean, standard deviation, minimum, and maximum values were determined using Excel 2019. SPSS 18.0 (IBM, Armonk, NY, USA) software was used to determine one-way ANOVA (*p* < 0.05 indicates a significant difference) and paired-sample *t*-tests. Box line plots were drawn using Origin 2021 (Lab, New York, NY, USA), and SIMCA-p13.0 software (Umetrics, Sartorius, Göttingen, Germany) was used for principal component analysis.

### 2.6. Chemometric Analysis

PLS-DA is a supervised pattern recognition method based on traditional PLS regression and a number of Y categorical variables for discriminant analysis. It is often used to deal with classification and discrimination problems. Banana samples were randomly divided into a training set (*n* = 93) and a test set (*n* = 30). PLS-DA was used to establish qualification models to identify banana origin. A total of 53 variables, including 4 stable isotopes and 49 elemental contents, were used as the input variables, and the geographical origin (HN or NHN) was used as the output variable. The model data set was divided and repeated 10 times, and the performance of the model was measured by the average classification accuracy. The variable importance of projection (VIP) was calculated to select the most important variables for model establishment (VIP > 1). The VIP calculation formula is shown below:

$$VIP_{j}=\sqrt{p\sum_{k=1}^{h}c_{k}^{2}w_{jk}^{2}/\sum_{k=1}^{h}c_{k}^{2}}$$

where VIP_j_ is the VIP value of the jth variable, *p* is the number of X-variables, k is the kth latent variable out of h latent variables, c_k_ is the regression weight of the kth latent variable, and w_jk_ is the jth element of the kth column of the weight matrix w for the X-variables.

For each division of the model data set, a group of elements with VIP > 1 was obtained, and the division was repeated 10 times to obtain 10 groups of elements with VIP > 1. From the 10 groups of elements, the overlapping elements were filtered out, and the final model was built using these overlapped elements. Chemometric models were performed in SIMCA-p13.0 software.

## 3. Results and Discussion

### 3.1. Stable Isotope and Multi-Elemental Characteristics of Hainan Bananas

**Figure 2:**
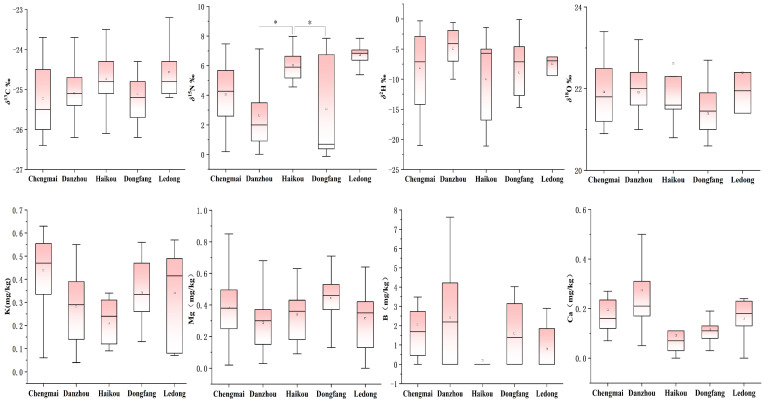
Box diagrams of stable C, N, H, and O isotopes, and K, Mg, B, and Ca contents of bananas from five Hainan cities in China. Note: * There were significant differences in representation.

A total of 75 banana samples were collected from five Hainan cities (Ledong, Chengmai, Danzhou, Haikou, and Dongfang). Isotope results showed that bananas from Danzhou, Haikou, and Dongfang had a wide range of *δ*^15^N values (*p* < 0.05). Bananas from Danzhou City had the lowest *δ*^15^N values of 2.7‰, and samples from Ledong County had the highest *δ*^15^N values of 6.2‰ (Figure 2). Bananas planted in Danzhou may be cultivated with chemical fertilizers, and samples planted in Ledong may use organic composts or animal manures, or may even have minimal fertilizer treatments [23]. The other three stable isotopes did not show significant differences due to the close geographical proximity and similar climatic influences of these five Hainan cities. The *δ*^13^C values ranged from −22.3‰ to −26.7‰ while the *δ*^2^H and *δ*^18^O values ranged from −30.1‰ to −16.5‰, and 18.9‰ to 28.8‰, respectively.

A total of 49 elements were determined in bananas from the five Hainan cities. There were no obvious elemental differences between samples from the five cities. The highest elemental contents in Hainan banana were potassium (K) with 10.54 mg/kg, followed by boron (B) with 9.70 mg/kg, magnesium (Mg) with 6.14 mg/kg, and calcium (Ca) with 3.22 mg/kg [24]. Ca content is an indicator of banana fruit quality and is important as it prevents fruit splitting, enhances sweetness, and increases the storage period [25]. Mean elemental B content was 1.87 mg/kg, and the wide range of values may be due to elemental differences in the soil, which affects its accumulation in plants [26]. There were no significant differences in the elemental contents of bananas across the five cities of Hainan (*p* > 0.05).

### 3.2. Differences Between Hainan and Non-Hainan Bananas

**Figure 3:**
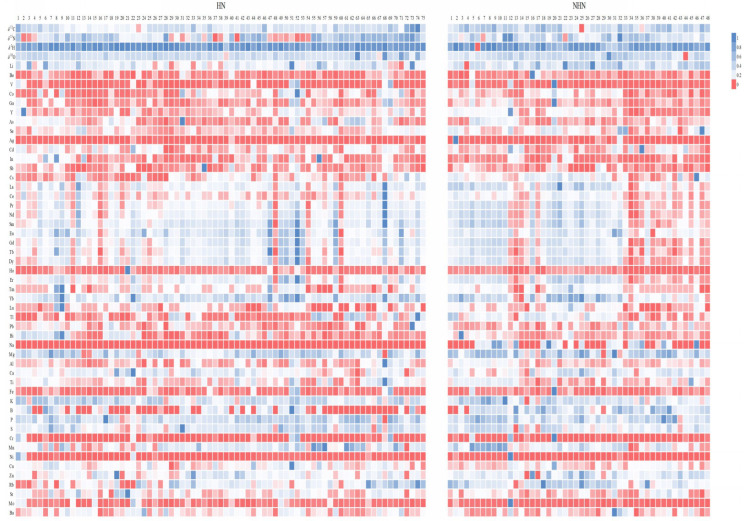
Heatmap of HN and NHN bananas in China using stable isotopes, multi-elements, and individual samples.

A heatmap summarizes the stable isotope and multi-element data of HN and NHN bananas (Figure 3). Colored squares on the heat map indicate normalized data (0–1) for stable isotopes and elemental contents of bananas (vertical axis) shown against banana origin (horizontal axis). Differences are seen within and between the HN and NHN datasets, identifying key origin-influenced variables.

A significant difference (*p* < 0.05) was found between the mean *δ*^13^C values of HN and NHN bananas (−25.1‰ and −24.3‰, respectively). While the range of HN bananas *δ*^13^C values (−26.7‰ to −22.2‰) was similar to NHN bananas (−27.0‰ to −22.3‰), the *δ*^13^C values of NHN bananas were significantly more positive than those of HN bananas. This may be attributed to the longer duration and higher intensity of sunshine in the Hainan region, resulting in higher carbon dioxide (CO_2_) uptake and lower CO_2_ respiration during photosynthesis. Bananas are a C_3_ plant [27] where the carbon isotope fractionation occurs via the Calvin cycle. Carbon dioxide from the air is absorbed by the plant during respiration to form sugars [22], resulting in more negative *δ*^13^C values than C4 plants. In addition, higher temperatures in the Hainan region increase the transpiration rate and water utilization efficiency of plants, which further enhances the enrichment of ^12^C during CO_2_ uptake [28].

The *δ*^15^N values of HN bananas ranged between −0.1‰ and 8.8‰, while NHN *δ*^15^N values spanned a range of 0.4‰ to 10.7‰. A significant difference was observed between the *δ*^15^N values of HN and NHN bananas (*p* < 0.05), with the former generally displaying lower values. Different *δ*^15^N values for different HN and NHN regions suggest a range of cultivation methods using synthetic, organic, or minimal fertilization practices [29]. Since the nitrogen in chemical fertilizers is of atmospheric origin, synthetic fertilizer *δ*^15^N values are closer to 0‰, the *δ*^15^N value of air, than organic fertilizers or natural soil nitrogen [30].

No significant *δ*^2^H and *δ*^18^O differences were seen between HN and NHN bananas. Often large *δ*^2^H and *δ*^18^O differences occur in plants affected by different precipitation events and fluctuating temperatures during growth [31], as plant *δ*^2^H and *δ*^18^O values are most affected by rainfall and transpiration, leading to different water fractionation effects [32]. Meteorological data from the HN and NHN regions of China in 2023 (Table S3) show that average temperatures, relative humidity, and precipitation during the banana growing season were relatively similar between origins.

According to Table 1, some HN banana variables were significantly different (*p* < 0.05) from NHN bananas, including significant differences in *δ*^13^C, *δ*^15^N, Na, Al, Ca, Ti, B, S, Co, Mn, Rb, Sr, Cd, Cs, Pr, Nd, Sm, Gd, Tb, Dy, Er, Yb, and Tl values, suggesting that they are effective in distinguishing between different banana origins. Previous research [33] showed that K, P, Ca, Mg, Mn, and Zn were the major elements present in bananas. The mean contents of these elements (K, Ca, B, Mg, Sr, Mn) were higher in bananas from the NHN regions of China than from the HN region, whereas the maximum contents of K, Ti, Fe, B, V, Li, Cr, Cu, Rb, As, Se, Ag, Sb, Cs, Ce, Tl, and Pb were much higher in HN bananas than in bananas from the non-Hainan region. The elemental compositions of plant tissues are influenced by soil composition, such as soil type, elemental content, pH, porosity, and humus content [34]. Elemental compositions of plants are therefore closely related to the soil geochemistry and plant–soil interactions. Hainan is a tropical island in China with Fe-rich soils and influenced by coastal climate effects (more frequent rain, higher temperatures, sea spray) compared to the other inland regions affecting the presence and abundance of elements found in bananas [35]. However, more research is required to link the soil elements to corresponding elements in bananas.

The non-Hainan regions of China (Yunnan, Guangxi, Guangxi, and Fujian) are geographically far apart, and the number of collected samples was relatively small, so it is difficult to aggregate the different variable characteristics for each non-Hainan region. Consequently, it is difficult to differentiate bananas from each of the non-Hainan regions. However, there were significant elemental differences between HN and NHN bananas, which helped to construct a traceability model to identify HN bananas.

### 3.3. Model Performance and Key Variable Selection of PLS-DA Model

PLS-DA was used to categorize the origin of 123 banana samples, which were divided into a training set (93 samples) and a test set (30 samples). A total of 53 variables were used to construct an initial PLS-DA origin model (4 stable isotopes and 49 elemental variables). The training and testing datasets were replicated ten times, resulting in ten model outcomes. Subsequently, VIP screening was performed on the ten replicates, selecting only variables with VIP > 1 for a second round of PLS-DA discrimination. Variables that were common across all ten replicates of the VIP data were identified and used to establish the final discriminant model. The reliability of the results was validated through 10 repetitions of training and testing set data, with the final results represented by the mean value of the 10 repetitions (Table S4a). The overall model accuracy for the training and test sets using 53 variables reached 83.7% and 84.6%, respectively. The discrimination ability for HN bananas reached 91.8%, indicating that the origin tracing model exhibited a robust predictive performance (Table 2).

**Figure 4:**
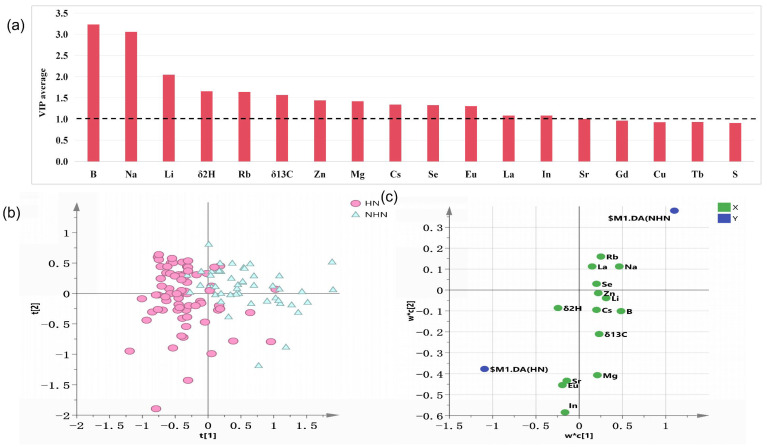
(**a**) VIP plot for 10 repetitions, (**b**) PLS-DA scores plot for HN and NHN origin bananas, (**c**) Loadings plot of the PLS-DA model.

A total of 14 variables (B, Na, Li, Rb, *δ*^2^H, Zn, *δ*^13^C, Mg, Cs, Se, Eu, La, In, and Sr) with VIP values > 1 were consistently present across the 10 repetitions (Figure 4a). These 14 key variables were used to establish the final model, and the discrimination results are shown in Table S4b. The optimized 14-variable model showed a higher predictive origin accuracy than the 53-variable model, indicating that it had a better classification performance. The overall accuracy of the training and test sets of the 14-variable model reached 87.3% and 90.9%, respectively. The predictive accuracy of the test set for the HN banana samples was 96.6%, which was a slight improvement compared to the 53-variable model, making it even more reliable (Table 2).

Figure 4 shows the scores and loadings plots of the final PLS-DA model. On the horizontal axis of the score plot, HN and NHN bananas are differentiated according to their origins, with a slight overlap. The loadings plot shows that HN origin bananas are mostly influenced by *δ*^2^H, Eu, Sr, and In, while NHN samples are typically characterized by B, Na, Li, Rb, Zn, *δ*^13^C, Mg, Cs, Se, and La.

While two previous banana studies used *δ*^13^C and *δ*^15^N isotope values to identify conventionally farmed bananas grown with synthetic fertilizers from organic bananas fertilized with organic manures from different countries [36,37], this origin verification research is the first study to undertake a regional characterization of Chinese bananas. The high accuracy rate of the models suggests that the combination of stable isotopes and elements is sufficient to reliably validate a traceability model for bananas from Hainan Province.

## 4. Conclusions

In this study, stable isotope and elemental stoichiometric PLS-DA models were used to successfully distinguish between Hainan and non-Hainan origin bananas, providing an effective method for tracing the origin of Hainan-grown bananas. The results show that banana samples from Hainan have unique isotopic and elemental fingerprints compared to other regions in China. Combined with PLS-DA, the discrimination model accuracy to correctly determine HN and NHN banana origin reached 90.9% using a 14-variable model. This model not only helps to identify and combat origin fraud but also provides a scientific basis for the protection of the Hainan banana brand, the economic development of the Hainan banana industry, and the stability of banana growers’ income.

## Figures and Tables

**Table 1:** Stable isotope and multi-element values for Hainan (HN) and non-Hainan (NHN) bananas in China.

Variables	Hainan Samples			Non-Hainan Samples			*p*
	Min	Max	Mean	Min	Max	Mean	
δ^13^C (‰)	−26.7	−22.2	−25.1	−27.0	−22.3	−24.3	0.001
δ^15^N (‰)	−0.1	8.8	3.6	0.4	10.7	3.9	0.019
δ^2^H (‰)	−16.5	30.0	6.1	−11.3	62.1	10.8	0.073
δ^18^O (‰)	18.9	28.8	22.0	18.2	28.3	22.0	0.627
Na (mg/kg)	0.00	0.09	0.01	0.00	0.07	0.01	0.010
Mg (mg/kg)	2.22	6.14	3.98	2.29	6.84	4.07	0.189
Al (mg/kg)	0.00	0.10	0.01	0.00	0.02	0.01	0.033
Ca (mg/kg)	0.48	3.22	1.18	0.56	4.26	1.33	0.018
K (mg/kg)	2.76	10.54	5.28	3.08	7.65	5.38	0.332
Ti (mg/kg)	0.02	0.29	0.06	0.02	0.11	0.04	0.036
Fe (mg/kg)	0.03	2.39	0.17	0.03	0.22	0.08	0.085
B (mg/kg)	0.00	9.70	1.87	0.00	8.50	2.16	0.031
P (mg/kg)	0.40	1.06	0.74	0.43	1.27	0.71	0.369
S (mg/kg)	0.23	0.85	0.41	0.22	0.68	0.35	<0.001
V (mg/kg)	0.00	0.53	0.03	0.00	0.18	0.01	0.388
Li (µg/kg)	0.00	258.45	74.04	10.53	197.70	74.89	0.113
Be (µg/kg)	0.15	49.55	2.52	0.15	10.68	1.66	0.735
Cr (µg/kg)	0.00	67.33	3.02	0.00	41.19	2.23	0.764
Co (µg/kg)	3.41	169.72	58.31	3.08	103.76	32.84	0.001
Mn (µg/kg)	3.41	169.72	47.73	3.08	143.06	49.38	0.045
Ni (µg/kg)	0.00	6.18	0.61	0.00	150.26	7.26	0.099
Cu (µg/kg)	0.85	31.52	4.74	1.95	12.41	5.06	0.846
Zn (µg/kg)	5.46	33.78	13.05	5.87	38.44	11.52	0.699
Ga (µg/kg)	0.50	24.60	2.36	0.28	7.15	1.42	0.117
Rb (µg/kg)	1.66	46.27	15.79	0.66	41.78	7.31	<0.001
Sr (µg/kg)	0.27	2.76	1.03	0.32	8.57	1.64	0.033
Y (µg/kg)	3.08	35.10	11.34	1.85	102.18	10.46	0.675
As (µg/kg)	0.45	62.43	7.20	0.00	18.60	5.10	0.163
Se (µg/kg)	1.15	60.25	7.21	0.00	42.23	7.21	0.221
Mo (µg/kg)	0.02	10.18	0.78	0.01	45.31	2.49	0.199
Ag (µg/kg)	0.00	0.42	0.01	0.00	0.08	0.00	0.401
Cd (µg/kg)	0.30	3.58	1.37	0.28	15.88	2.95	0.001
In (µg/kg)	0.00	12.75	0.71	0.05	10.75	1.18	0.459
Sb (µg/kg)	0.00	62.10	4.16	0.00	6.78	2.27	0.051
Cs (µg/kg)	2.10	141.53	22.30	0.28	66.58	9.10	0.002
Ba (µg/kg)	0.39	5.08	1.40	0.30	12.75	2.17	0.066
La (µg/kg)	1.48	24.98	7.03	0.73	20.88	4.77	0.082
Ce (µg/kg)	1.95	44.95	6.65	0.95	23.33	5.54	0.412
Pr (µg/kg)	0.20	4.23	1.08	0.18	2.30	0.65	0.005
Nd (µg/kg)	1.13	16.13	4.61	0.70	11.50	2.73	0.001
Sm (µg/kg)	0.18	2.90	0.94	0.08	2.33	0.54	<0.001
Eu (µg/kg)	0.10	1.05	0.30	0.03	0.83	0.29	0.425
Gd (µg/kg)	0.25	4.53	1.27	0.15	3.15	0.74	<0.001
Tb (µg/kg)	0.05	0.68	0.18	0.03	0.50	0.11	0.005
Dy (µg/kg)	0.15	3.43	0.92	0.10	1.98	0.51	<0.001
Ho (µg/kg)	0.03	0.63	0.19	0.03	6.38	0.25	0.655
Er (µg/kg)	0.10	1.73	0.48	0.05	0.90	0.28	0.002
Tm (µg/kg)	0.00	0.33	0.06	0.00	0.48	0.05	1.000
Yb (µg/kg)	0.03	0.70	0.30	0.00	0.73	0.20	0.002
Lu (µg/kg)	0.00	0.30	0.05	0.00	0.53	0.05	0.638
Tl (µg/kg)	0.43	83.38	15.69	0.00	19.03	3.43	<0.001
Pb (µg/kg)	8.98	559.23	52.28	9.50	252.48	61.49	0.696
Bi (µg/kg)	0.00	5.90	0.43	0.00	5.68	0.80	0.133

Note: *p* indicates significant differences between Hainan and non-Hainan bananas in China for different elements (*p* < 0.05).

**Table 2:** PLS-DA model accuracy results (%) for HN and NHN bananas in China.

Data	Index	Model 1 (53 Variables)	Model 2 (14 Variables)
**Training set**	HN samples	90.6	92.7
	NHN samples	72.5	78.8
	**Overall accuracy**	**83.7**	**87.3**
**Test set**	HN samples	91.8	96.6
	NHN samples	71.8	82.4
	**Overall accuracy**	**84.6**	**90.9**
**All**	HN samples	90.9	93.7
	NHN samples	72.3	79.7
	**Overall accuracy**	**83.9**	**88.2**

## Data Availability

The data presented in this study are available on request from the corresponding author.

## Supplementary Materials

The following supporting information can be downloaded at: https://www.mdpi.com/article/10.3390/foods14040554/s1, Figure S1: Image of Hainan bananas; Table S1: Information on China’s banana industry from five provinces; Table S2: Sample information of bananas collected from different origins; Table S3: Meteorological data of for Hainan and non-Hainan regions of China in 2023; Table S4: PLS-DA predictive accuracy results of ten training and testing set repetitions to model banana origin for HN, NHN, and all sample locations using (a) a 53-variable model and (b) a 14-variable model.
