# Supplementary material for: Application of Stable Isotopes and Multi Elemental Fingerprints to Verify the Origin of Premium Chinese Hainan Bananas
Source: Foods. 2025 Feb 7;14(4):554. doi: 10.3390/foods14040554 (PMC11853982; doi:10.3390/foods14040554)
Supplement: Supplementary file 1 [file foods-14-00554-s001.zip › foods-3455501-supplementary.pdf]

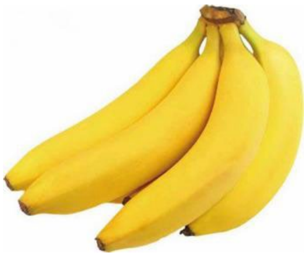

Figure S1. Image of Hainan Bananas.

Table S1. Information on China’s banana industry from 5 provinces.

Banana Variety	Planting Province	Planting Area (hectares)	Yield (tons)
Baodao Banana	Hainan	34900	1285800
Guijiao No. 6	Guangxi	62400	1073400
Xiangya Banana	Guangdong	111300	4000000
Tianbao Banana	Fujian	3000	900000
Williams Banana	Yuannan	5333.33	110000

Table S2. Sample information of bananas collected from different origins.

Origin	Province	City	Samples
Hainan	Hainan	Chengmai	23
		Danzhou	25
		Haikou	7
		Dongfang	14
		Ledong	6
Non-Hainan	Guangdong	Guangzhou	4
		Maoming	2
		Zhanjiang	3
	Yunnan	Honghe Hani & Yi Autonomous Prefecture	7
		Xishuangbanna Dai Autonomous Prefecture	8
		Pu'er	1
		Dehong Dai & Jingpo Autonomous Prefecture	1
		Yuxi	2
	Guangxi	Naninng	6
		Bose	3
	Fujian	Zhangzhou	11

Table S3. Meteorological data of for Hainan and non-Hainan regions of China in 2023.

**Origin**		**T (℃)**	**Tg (℃)**	**RHU (%)**	**RHUg (%)**	**PRE (mm)**	**PREg (mm)**	**SSD (h)**	**SSDg (h)**
Hainan	Hainan	25.4	28.6	84.0	88.4	1639.0	1785.0	2200.0	2030.0
Non-Hainan	Yunnan	18.1	19.4	71.0	77.0	887.2	795.8	2025.7	1945.6
	Guangdong	22.7	23.6	77.0	80.0	1818.9	1546.4	1745.8	1573.8
	Guangxi	25.1	26.6	76.0	79.0	1887.6	1694.8	1607.0	1467.2
	Fujian	20.5	22.1	82.0	85.0	1621.3	1452.5	1824.0	1665.0
**Average value**		**21.6**	**22.9**	**76.5**	**80.2**	**1553.7**	**1372.3**	**1800.6**	**1662.9**

Note: T: Mean annual temperature, Tg: Average annual temperature during growing season, RHU: Mean annual relative humidity, RHUg: Mean relative humidity during growing season, PRE: Annual precipitation, PREg: Annual precipitation during growing season (from April to October), SSD: Sunshine duration, SSDg: Sunshine duration during growing season (from April to October).

Table S4. PLS-DA predictive accuracy results of ten training and testing set repetitions to model banana origin for HN, NHN and all sample locations using (a) a 53-variable model, and (b) a 14-variable model.

**(a)**	**Data Division**	**HN Accuracy (%)**	**NHN Accuracy (%)**	**ALL Accuracy (%)**
Training (75%)	1	89.5	89.5	89.5
	2	93.0	63.9	81.7
	3	88.5	75.0	83.9
	4	96.5	66.7	85.0
	5	86.0	72.2	80.7
	6	91.2	74.3	84.8
	7	93.0	72.2	85.0
	8	91.1	75.7	85.0
	9	89.5	75.0	83.9
	10	87.9	75.0	83.0
	**Average**	**90.6**	**72.5**	**83.7**
Test (25%)	1	88.9	83.3	86.7
	2	88.9	83.0	86.7
	3	88.9	75.0	83.3
	4	83.3	84.6	83.9
	5	100.0	58.3	83.3
	6	95.5	50.0	81.3
	7	88.9	83.3	86.7
	8	94.1	83.3	89.7
	9	95.5	50.0	81.3
	10	94.4	66.7	83.3
	**Average**	**91.8**	**71.8**	**84.6**
	**All Average**	**90.9**	**72.3**	**83.9**

**(b)**	**Data Division**	**HN Accuracy (%)**	**NHN Accuracy (%)**	**ALL Accuracy (%)**
Training (75%)	1	92.5	84.2	89.0
	2	93.0	75.0	86.0
	3	93.0	75.0	86.0
	4	93.0	80.6	88.2
	5	93.0	80.6	88.2
	6	93.0	77.8	87.1
	7	91.2	77.8	86.0
	8	91.2	77.8	86.0
	9	92.5	84.2	89.0
	10	94.6	75.0	87.0
	**Average**	**92.7**	**78.8**	**87.3**

Test (25%)	1	100.0	83.3	93.3
	2	100.0	91.7	96.7
	3	94.4	83.3	90.0
	4	94.1	83.3	89.7
	5	100.0	75.0	90.0
	6	88.9	83.3	86.7
	7	100.0	75.0	90.0
	8	94.1	83.3	89.7
	9	100.0	81.8	93.3
	10	94.4	83.3	90.0
	**Average**	**96.6**	**82.4**	**90.9**
	**All Average**	**93.7**	**79.7**	**88.2**
